# Raman scattering owing to magneto-polaron states in monolayer transition metal dichalcogenides

**DOI:** 10.1038/s41598-024-63179-5

**Published:** 2024-06-04

**Authors:** C. Trallero-Giner, D. G. Santiago-Pérez, D. V. Tkachenko, G. E. Marques, V. M. Fomin

**Affiliations:** 1 Departamento de Física, Universidade Federal de São Carlos, São Carlos, São Paulo 13.565-905 Brazil; 2 Universidad Autónoma del Estado de Morelos, Ave. Universidad 1001, 62209 Cuernavaca, Morelos Mexico; 3 Pridnestrovian State University, 25 October Str., 128, 3300 Tiraspol, Republic of Moldova; 4 Institute for Emerging Electronic Technologies (IET), Leibniz Institute for Solid State and Materials Research (IFW) Dresden, Helmholtzstraβe 20, 01069 Dresden, Germany; 5 Faculty of Physics and Engineering, Moldova State University, Str. A. Mateevici 60, 2009 Chişinău, Republic of Moldova

**Keywords:** Condensed-matter physics, Quantum physics

## Abstract

Magneto-optical measurements are fundamental research tools that allow for studying the hitherto unexplored optical transitions and the related applications of topological two-dimensional (2D) transition metal dichalcogenides (TMDs). A theoretical model is developed for the first-order magneto-resonant Raman scattering in a monolayer of TMD. A significant number of avoided crossing points involving optical phonons in the magneto-polaron (MP) spectrum, a superposition of the electron and hole states in the excitation branches, and their manifestations in optical transitions at various light scattering configurations are unique features for these 2D structures. The Raman intensity reveals three resonant splittings of double avoided-crossing levels. The three excitation branches are present in the MP spectrum provoked by the coupling of the Landau levels in the conduction and valence bands via an out-of-plane $A_1$-optical phonon mode. The energy gaps at the anticrossing points in the MP scattering spectrum are revealed as a function of the electron and hole optical deformation potential constants. The resonant MP Raman scattering efficiency profile allows for quantifying the relative contribution of the conduction and valence bands in the formation of MPs. The results obtained are a guideline for controlling MP effects on the magneto-optical properties of TMD semiconductors, which open pathways to novel optoelectronic devices based on 2D TMDs.

## Introduction

Resonant Raman scattering in a magnetic field *B* is a powerful nondestructive tool to study the electron-hole pair (EHP) energy spectrum in transition metal dichalcogenide (TMD) semiconductors^1^. Recently, a new series of magneto-polaron resonances (MPRs) in monlayer (ML) TMD materials was reported^2^. The coupling of the two Landau levels with the optical phonons leads to the appearance of three excitation branches. This effect should strongly impact the magneto-optical properties, in particular, those related to magneto-resonant Raman spectroscopy (MRRS)^3^. The first observation and explanation of the existence of excited states in the magneto-resonant Raman scattering were reported in InP^4,5^. It was shown that renormalized Landau levels exhibit a ladder-like structure coupling the excited states via the electron-phonon interaction (EPI), as found experimentally. By adjusting the laser energy and the field *B*, Raman spectroscopy allows for revealing the fundamental differences between bulk systems and 2D structures. The reported series of MPRs in TMDs raise new fundamental questions on the resonant magneto-polaron Raman scattering (RMPRS), where a strong anticrossing between Landau levels can occur in the conduction and valence bands. Hence, it is imperative to address the role of the EPI in the RMPRS, the relative contribution of scattering intensities in different scattering configurations, and how the Raman spectrum results in the range of *B*, where both electron and hole states can resonate via an optical phonon. Straight-forward analytical models that describe RMPRS in the TMD materials are necessary for understanding Raman measurements. These allow us to determine conditions of the MP resonance and to acquire hitherto inaccessible information about the strength of the EPI and the band structure of the TMD materials. Since the magnetic field is efficient for tuning the light-matter interaction in ML TMDs, the analyzed RMPRS open perspectives for creation of new optoelectronic devices ranging from lasers^6,7^ and light emitters^8^, through frequency converters^6^, modulators^7,8^, and detectors^9^ to plasmonic generators^6^ and sensors^6^. The fundamental findings of the present work are the characterization and the quantitative assessment of the parameters governing the effects of MP on the first-order resonant Raman scattering in ML TMDs.

## Results

**Figure 1:**
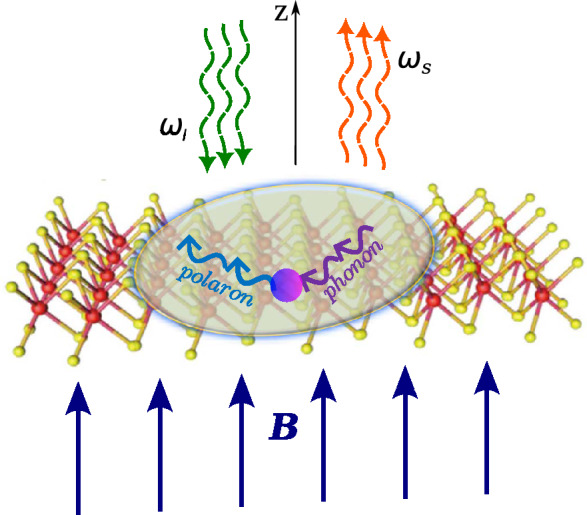
Scheme of the RMPRS in the Faraday configuration in a ML TMD. The incoming light with the frequency $\omega _l$ and the momentum $\kappa _l$ creates an EHP in an external magnetic field *B*. If the energies of the two electronic states are coupled by the phonon field, a *magneto-polaron* quasi-particle occurs. Finally, the resonant MP quasi-particle annihilates with emitting a scattered photon with the frequency $\omega _s$ and the momentum $\kappa _s$.

We investigate the RMPRS in 2D TMDs at the $K$ $( K ^{'})$-point of the Brillouin zone (BZ) (see Fig. 1), assuming the photon excitation energy $\hbar \omega _{l}$ well above the gap energy, $E_g$, and high field *B*, when the scattering is ruled by the uncorrelated electron-hole pair (EHP). When the energies of two Landau levels are separated by the energy of one optical phonon, the MP resonance takes place^10^. The resonant effect is due to EPI that lifts the degeneracy of the involved Landau levels. At a certain critical magnetic field, $B_c$, two Landau levels $N_1$ and $N_2$ are coupled by the phonon frequency, therefore the polaron effects should be detected in the light dispersion. The resonances at $B_c$ between different Landau levels via one phonon raise questions about how MPRRS actually occurs. It is clear that, for a realistic treatment of Raman scattering, it is necessary to know the renormalized spectrum of the EHP in an applied field *B* due to the electron interaction with the phonon field.

The Raman efficiency in a 2D TMD is defined as^11^

$$\begin{aligned} \frac{dI}{d\Omega }=\frac{1}{S}\int \frac{\partial ^2 \sigma }{\partial \Omega \partial \omega _s} d\omega _s,\;\; \text {with}\;\;\; \frac{\partial ^2\sigma }{\partial \Omega \omega _s}=\frac{V^2}{4\pi ^2\hbar c^4}\frac{ \omega _s^3\eta _l\eta _s^3}{\omega _l} \left| W_{\text {FI}}\right| ^{2}\delta (\hbar \omega _l-\hbar \omega _s-\hbar \omega _o), \end{aligned}$$

where *S* is the normalization area, $dI/d\Omega$ is a dimensionless quantity, $\partial ^2 \sigma /(\partial \Omega \partial \omega _s)$ is the scattering cross-section in a volume $V=Sb$ (*b* is the sample width) per unit solid angle $\Omega$ and per unit scattered light frequency $\omega _{s}$, $\eta _l(\eta _s$) is the refractive index for the incoming (scattered) light, *c* is the speed of light in the vacuum, $W_{\text {FI}}$ is the scattering amplitude from the initial state *I* of the system in the presence of the incident photon $\omega _l$ to the final state *F* with a scattered photon of frequency $\omega _s$ and a phonon $\omega _o$.

At the resonance, the main contribution to the scattering amplitude $W_{\text {FI}}$, considering the MP effects (see Fig. 1) it is written down as

1 $$\begin{aligned} W_{\text {FI}}= &  \sum \limits _{\{\mu _1\},\{\mu _2\}}\left\langle F\right| \hat{H}_{E-R}^{^{(\text {+})}}({\varvec{\sigma }}^\pm )\left| \Psi _{\mu _2}\right\rangle G_{\{\mu _2\}}(\hbar \omega _l-\hbar \omega _o) \left\langle \Psi _{\mu _2}\right| \hat{H} _{E-P}\left| \Psi _{\mu _1}\right\rangle \nonumber \\\times &  G_{\{\mu _1\}}(\hbar \omega _l) \left\langle \Psi _{\mu _1}\right| \hat{H}_{E-R}^{^{(\text {-})}}({\varvec{\sigma }}^\pm )\left| I \right\rangle , \end{aligned}$$

where $\hat{H}_{E-P}$ and $\hat{H}_{E-R}^\pm$ are the EPI and the electron-radiation Hamiltonians, $\Psi _{\mu _i}$ ($i=1,2$) are the intermediate EHP Landau states, $\mu =\mu (N_e, k_{ey};N_h, k_{hy})$ represents the set of the EHP states with the Landau quantum numbers $N_e$, $N_h$ and the wave vectors $k_{ey}$, $k_{hy}$, $G_{\{\mu \}}$ is the Green’s function at $T=0$ K considering the MP quasi-particle. The summation in Eq. (1) is carried out over the MP virtual intermediate states {$\mu _i$} (*i* = 1, 2), the coupling between the free EHP state $\left| \mu \right\rangle$ with the phonon field provides a new set of quantum numbers, i.e $\left| \mu \right\rangle \rightarrow \left| \{\mu \}\right\rangle$ (see Supplementary Information). A zero-order correction is assumed for the vertices that correspond to the electron-radiation Hamiltonian and the EPI^12,13^.

### Resonant magneto-polaron Raman efficiency

**Figure 2:**
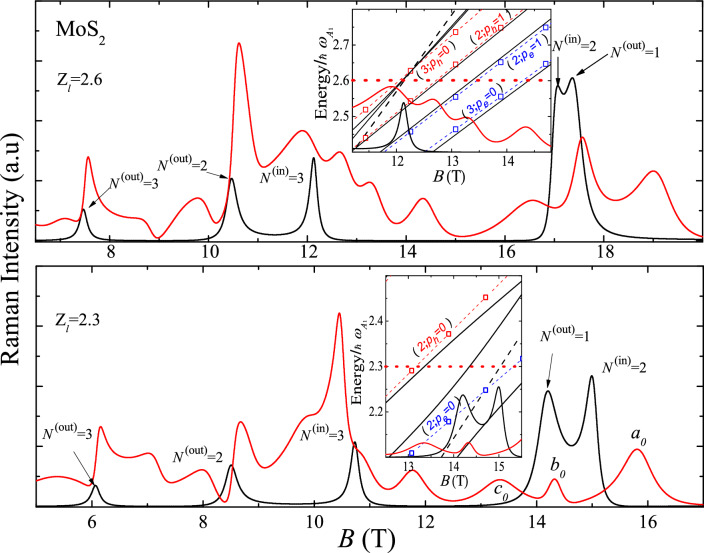
Profile of the resonant MP Raman efficiency (red solid lines) at the relative laser frequencies $Z_l=(\hbar \omega _{l}-E_g)/\hbar \omega _{A_1}$= 2.3 and 2.6 ($\hbar \omega _l$= 1.964 and 1.979 eV) for MoS$_2$. Black solid lines show the Raman scattering without the MP effects. The incoming and outgoing resonances for the bare Landau level *N* are indicated with black arrows. Insets represent the range of the MP spectra contributing to the Raman intensities: $Z_l$ =2.6, 11 T < *B* < 15 T and $Z_l$ = 2.3, 12.5 T < *B* < 15.5 T. Red (blue) dashed lines are guide for the eye, the value of $Z_l$ is indicated by a red doted line. The bare exited states are labeled by (*N*; $p_i$) ($i = e, h$), $a_0$, $b_0$ and $c_0$ denote the peaks related to the anticrossings at *N* = 2 (see the text).

**Figure 3:**
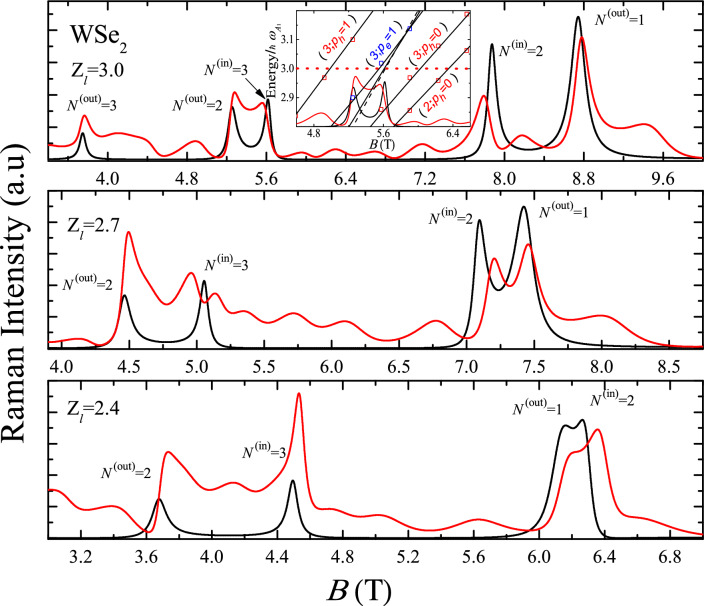
The same as Fig. 2 at $Z_l$=2.4, 2.7 and 3.0 ($\hbar \omega _l=$ 1.812, 1.822, 1.831 eV) for WSe$_2$. Inset demostrates the MP spectra as a function of *B* contributing to Raman intensities for $Z_l$=3.0 and 4.6 T < *B* < 6.5 T (see the text).

In the case of backscattering from a surface *S* in ML TMD in the Faraday configuration, where ***B*** $\parallel$ $\hat{z}$, $\kappa _{light}$ $\parallel$ ***B*** (see Fig. 1), the Raman selection rules provide that the light scattering is mediated by the short-range $A_1$(ZO)-homopolar deformation potential (DP) and the long-range Pekar-Fröhlich (PF) electron-phonon interactions^14,15^. Employing Eq. (1), collecting the Eqs. (S1) and (S2) and considering the MP spectrum displayed in the Supplementary Information S2, the Raman scattering intensity due to the intravalley-DP interaction for the $A_1$(ZO)-mode, is

2 $$\begin{aligned} \frac{dI^{\text {DP}}}{d\Omega } = I_0l_c^{-4}\left| \sum _{n(N)}\frac{1}{\left[ \hbar \omega _l-E_g-E_{n(N)}(B)-i\delta \right] \left[ \hbar \omega _l-E_g-\hbar \omega _{A_1}-E_{n(N)}(B)-i\delta \right] }\right| ^2\;, \end{aligned}$$

where

3 $$\begin{aligned} I_0 = \left( \frac{\omega _s}{\omega _l}\right) ^2\frac{\eta _s^2}{\pi ^4c^4}\frac{e^4a^4t^4}{\hbar ^2}\frac{\left( D_c-D_v\right) ^2}{2\rho _m\hbar \omega _{A_1}}\;, \end{aligned}$$

and $E_{n(N)}$ is the renormalized EHP energy given by Eq. (4). For fixed values of *N* and ${ B}$, in the sum above, the “dressed” states must be added: (i) the Landau ground state plus (ii) the excited states of the electron and hole with indexes $p_e$, $p_h$= 0, 1,..., $N-1$. For the case of PF matrix element as given in (see Supplementary Information S1, Eq. (S3)), the in-plane phonon wave vector ${\varvec{q}}={\varvec{0}}$. Therefore, the scattering intensity $dI^{\text {LO}}/d\Omega$ is zero, i.e. the long-range interaction mechanism in a strictly 2D system is not active. Invoking a deviation from the normal incidence of light^11^, in the presence of impurities or defects^16^, the ${\varvec{q}}={\varvec{0}}$ selection rule is lifted, which leads to the light scattering via an LO-phonon mode. In the present treatment, we are focusing on strict backscattering configurations on the surface *S*. The dependence of the scattering efficiency in Eq. (2) on *B* for two and three values of the dimensionless laser energies $Z_l=(\hbar \omega _{l}-E_g)/\hbar \omega _{A_1}$ for MLs of MoS$_2$ and WSe$_2$, is displayed in Figs. 2 and 3, respectively. The insets show the regions of the polaronic spectrum contributing to the Raman intensities. The intersections of the excitation energy $Z_l$ (black dotted lines) with the relative energies $\hat{\epsilon }_{n(N)}/\hbar \omega _{A_{1}}$ of the EHP MP spectrum, determine the resonance peaks in the Raman profile. The uncoupled electron and hole excited states are labeled by (*N*; $p_i$), $(i=e,h)$.

**Figure 4:**
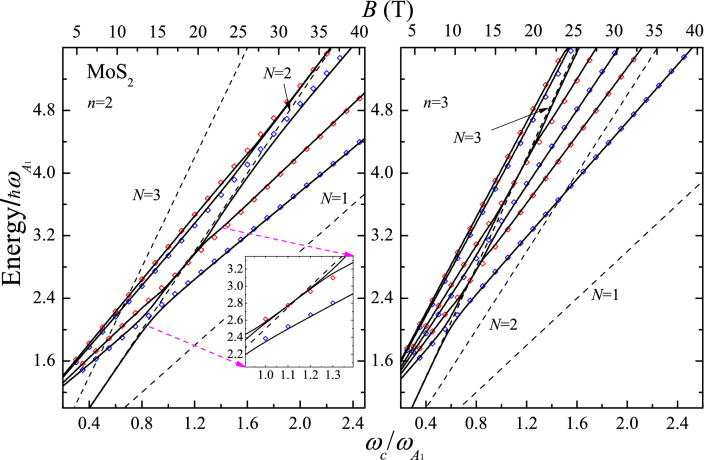
EPH MP spectra (solid black lines) of a ML of MoS$_2$ as a function of the relative cyclotron frequency $\omega _c/ \omega _{A_{1}}$ for the quantum numbers *n* = 2 and 3. The renormalized Landau levels are obtained taking $Re \{E\}$ in Eq. (4). The black dashed lines represent the bare Landau level *N*, the blue and red diamonds display the bare excited states for the electron $\hbar \omega _{e}^{(exc)}(N,p_e)$ and hole $\hbar \omega _{h}^{(exc)}(N,p_h)$ with $p_e$, $p_h$ = 0, 1, 2. The inset illustrates the contribution of the hole. In the calculation, the values of $\alpha _{\text {DP}}^{(\text {EH})}$ = $\alpha _{\text {DP}}m_e/\mu _{\text {EH}}$ = 0.0323 ($\mu _{\text {EH}}$ being the EH reduced mass), $\delta$ = 1 meV, $D_c$ = 5.8 eV/Å^19^ and $D_v$= 4.6 eV/Å^20^ are used.

**Figure 5:**
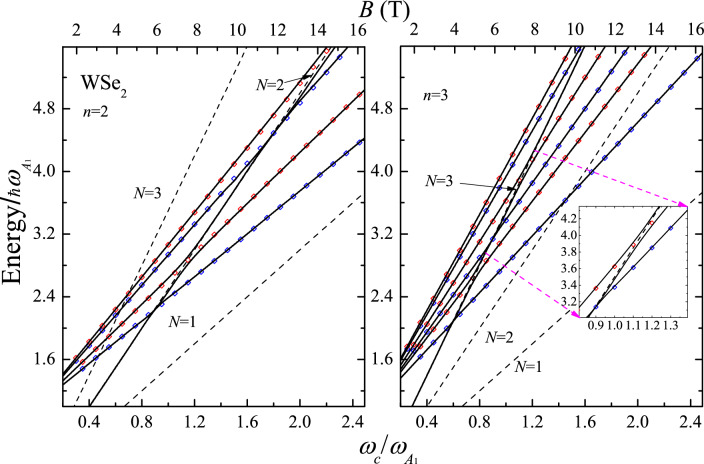
The same as Fig. 4 for a ML of WSe$_2$. The inset shows the second avoided crossing point for the valence band at $\omega _c/ \omega _{A_{1}}$ $\approx$ 1.2. The values of $\alpha _{\text {DP}}^{(\text {EH})}$= 0.0044, $\delta$= 1 meV, $D_c$= 2.3 eV/Å and $D_v$= 3.1/ÅeV^20^ are used for the $V_1$ $\rightarrow$ $C_1$ optical transition.

In the calculation, we consider the $\overline{Z}({\varvec{\sigma }}^-,{\varvec{\sigma }}^-)Z$ backscattering configuration at the $K$-valley of the BZ. For MoS$_2$, the main contribution corresponds to the valence band $V_1$ and the conduction band $C_2$ with the effective masses $m_{h(v_1)}/m_0$ = 0.54, $m_{e(c_2)}/m_0$ = 0.43^17^, $\hbar \omega _{A_1}$ = 49.5 meV^14^, and $E_g$= 1.85 eV^18^, while for WSe$_2$, the main contribution correspond to the interband allowed optical transitions $V_1\leftrightarrow C_1$ with $m_{h(v_1)}/m_0$= 0.36, $m_{e(c_1)}/m_0$= 0.28^17^, $\hbar \omega _{A_1}$ = 30.2 meV^14^, and $E_g$ = 1.74 eV^18^. For simplicity and without loss of generality, the Zeeman splitting is neglected. In this case, for the Mo (W) family, the difference of the Raman scattering intensity in the resonance ranges between 10 and 13 percent (4 and 6 percent) for the *N* = 2 Landau level. These ranges scale with the factors of 0.71 and 0.56 for *N* = 3 and 4. The Raman efficiency, in the backscattering configurations with circular polarization, and especially, the incoming and outgoing resonances, correspond to different spin orientations and optical transitions in the $K$- and $K^{'}$-valleys. These measurements provide the EH *g*-factor and information on the band parameters as a function of *B*. For the electronic virtual intermediate states in the Eq. (2), the presence of EHP intravalley transition is assisted by the $A_1$-phonon, where anticrossings occur between the Landau level and the excited states of the electron or hole at different magnetic fields. These avoided-crossing levels give rise to the three excitation branches, as demonstrated in Figs. 4 and 5 for the MP spectra of MoS$_2$ and WSe$_2$, respectively. In our results, the more realistic life-time broadening, $\Gamma _{n(N)}(B)$ as a function of *B*, occurring from a solution of the Dyson’s equation, is taken into account for each renormalized state with the energy $\hat{\epsilon }_{n(N)}$ (see Section S2 in Supplementary Information, where the effect of the broadening dependent on *B* in ML MoS$_2$ and WS$_2$ on the dressed Landau level *N* = 2 and four renormalized excited states with $p_e$ and $p_h$ = 0, 1 is shown). In Figs. 2 and 3, several regions are represented, where the MP effects determine the characteristics of the Raman intensity.

## Discussion

According to Eq. (2), Raman profiles provide insights into the EPH MP spectrum of each material. The relative contributions of the conduction and valence bands to the RMP coupling can be discerned from the Raman profile, depending on the exciting laser energy and the magnetic induction *B*. At MP resonances, each bare Landau level gives rise to three resonance lines. For illustration, consider the Raman profile of MoS$_2$ at $Z_l$=2.3, where three peaks (labeled as *a*$_0$, *b*$_0$, and *c*$_0$) are associated with the avoided crossing point at $\omega _{c_e}$($B\sim 16$ T)=$\omega _{A_{1}}/2$. The inset of the figure displays the excited states with $p_e$ = $p_h$ = 0 occurring at the MP *n* = 2 level. In all instances, characteristic shifts of polaronic states relative to the bare Landau level are evident. However, the three resonant branches exhibit distinct features depending on the excitation energy $\hbar \omega _l$ and the range of *B*. As depicted in Fig. 3, the anticrossing with $N = 3$ is masked by the simultaneous presence of the outgoing resonance from the $N^{(\text {out})}=2$ level and the renormalized hole excited state ($N=2$; $p_h=1$). For each MP state with the energy $\hat{\epsilon }_n(B)$, two resonance peaks occur in the Raman profile at $\hbar \omega _l-E_g$ = $\hat{\epsilon }_{n(N)}(B)$ and $\hbar \omega _s=\hbar \omega _l-\hbar \omega _{A_1}$ for the incoming and outgoing resonances, respectively. This phenomenon is clearly illustrated for both materials in Figs. 2 and 3, highlighting the distinct sets of incoming and outgoing resonances for the *n* = 2, 3 MP states. In Fig. 2, at $Z_l$=2.3, the electron contribution to the MP spectrum of MoS$_2$ becomes evident for *B* values corresponding to the vicinity of the Landau level *N* = 2, as illustrated in the inset of Fig. 2. Equation (5) reveals that the MP states arise from the avoided crossing point at $\omega _{c_e}$($B\sim 16$ T)=$\omega _{A_{1}}/2$ (refer to the inset of Fig. 4). Similarly, for WSe$_2$, caculations at $Z_l$=3.0 indicate that for $\omega _{c_h}$($B\sim 7$ T)=$\omega _{A_{1}}/2$ and $\omega _{c_h}$($B\sim 5$ T)=$\omega _{A_{1}}/3$, the valence band predominantly contributes to the polaronic quasi-particles. This is a consequence of the reconstruction of states *N* = 3 and *N* = 2 due to the EPI, as elaborated in Fig. 5, which shows the *n* = 2 and *n* = 3 components of the MP spectrum.

Importantly, the obtained results allow for the determining parameters of the band structure dominating the Raman efficiency. For MoS$_2$, the largest gaps of the MP states are provoked by the conduction band, while for WSe$_2$ those are attributed to the valence band. These effects are transferred to the sequence of peaks in the resonant profiles, which are linked to the $\hat{\epsilon }_{n(N)}$ energies. The sequence of resonances is a function of the effective masses $m_e$, $m_h$ and the deformation potentials $D_c$, $D_v$ (see Eq. (4)). Employing Eq. (3), we find that the relative contribution of the electron-hole-phonon interaction to the Raman intensity for MoS$_2$ and WSe$_2$ is *R* = 1.7. In the calculation, the values of the lattice constant *a*= 3.1635 Å (3.2954 Å) and the frequency $\omega _{A_1}$ = 399.29 cm$^{-1}$ (243.83 cm$^{-1}$) are used for MoS$_2$ (WSe$_2$). ^14^ This result implies that the MP effects are stronger in MoS$_2$ than in WSe$_2$. It is clearly manifested in the EHP MP spectra displayed in Figs. 4 and 5 and, consequently, in the Raman intensities for MoS$_2$ and WSe$_2$ (compare Figs. 2 and 3). The relative contribution of the electrons or holes to the selfenergy *S*(*E*, *N*) (see Eq. (S7)) is ruled by the ratio $D_v/D_c$. Therefore, the gaps occurring at each crossing point are associated with the peak positions determined by the conduction or the valence bands if $D_v/D_c$ <1 or $D_v/D_c$ >1, respectively. This fact allows for determining the band parameters, in particular, the optical DP, for which the reported values in the literature show a broad dispersion. ^18–20^

At the avoided crossing point, the sequence of three excitation branches in MoS$_2$ is as follows: the Landau level *N* plus two associated excited states, (*N*; $p_e$) for the conduction band and (*N*; $p_h$) for the valence band, in this order. For WSe$_2$, there is exchange between the electron and hole, i.e. (*N*; $p_e$) $\Leftrightarrow$ (*N*; $p_h$). This fact is a consequence of the DP values for the two materials. Finally, for laser energies below the bare excited states $\hbar \omega _{e}^{(exc)}(N;p_e)$ and $\hbar \omega _{h}^{(exc)}(N;p_h)$, the magneto-Raman scattering efficiency without the MP contribution is recovered, i.e., outside of the range of the MP spectrum. For comparison and discussion, the limit of a negligible polaron coupling is shown in Figs. 2 and 3 by solid black lines. Comparing both cases, with and without the polaronic coupling, reveals a rich structure derived when a MP quasiparticle is present. Not only the three MP branches, but also the renormalized excited states are present in a wide range of *B*. Moreover, those incoming and outgoing resonances, associated with the renormalized states (*N*; $p_e$) and (*N*; $p_h$), remain well-defined in the resonance profiles. Their relative intensities and peak positions depend on the excitation energy and the applied field *B*.

## Conclusions and outlook

We have provided an explicit expression for the resonant MP Raman scattering via the short-range DP EPI of a ML TMD. It is shown that the Raman scattering is prohibited in the parallel polarization for the 2D PF Hamiltonian. The essential features of MP quasiparticles and the role of the EHP on the spectra are studied. The resonant polaron coupling occurs through the $A_1$-DP mechanism. Optical phonons break the degeneracy between the EHP Landau levels and the bare electron ($\omega _e^{exc}$) and hole ($\omega _h^{exc}$) excited states, giving rise to three resonant branches in the renormalized energy spectrum. There exist two different sets of cyclotron frequencies due to the conduction and valence bands described by Eq. (5). The central results of these contributions are summarized in Figs. 4 and 5, where MP gaps, the splittings of double avoided-crossing levels in three levels, and the relative contribution of the valence or conduction bands to the RMPRS as a function of *B* are deduced. These studies are based on the reported MP complex-valued energy $E_{n(N)}$ as given by Eq. (4). In the polaron range, the incoming or outgoing resonances split into triplets (see Figs. 2 and 3). The amplitude and width of the upper, intermediate and lower branches mimic the renormalized complex energy $E_{n(N)}(B)$. Scanning the excitation light $\omega _l$ and *B*, EHP fan plots are derived from the Raman measurements, after excitonic correction (see Refs.^4,21^), allowing for a direct comparison with the theoretical predictions (4). The RMPRS, described by Eq. (2), provides a simple experimental alternative that allows for characterizing the excitation branches reported here as a function of *B*. In addition, further interesting studies of the light scattering processes can be carried out, such as, extension of the present model to the transitions between valleys (*K* $\leftrightarrow$ $K^{'}$) assisted by the acoustic-phonon modes. Here, a second-order Raman scattering takes place between two different EHP MP states in different valleys: the renormalized EHP energies $E_n$(*K*), $E_m$($K^{'}$) at the *K*- and $K^{'}$-points of the BZ are coupled by the LA or TA modes.

Finally, our results are of key importance for a wide range of magneto-optical effects, where interband optical transitions take place, as, for example, magneto-optical absorption^22^, magneto-reflectance^23^, and magneto-hot-luminescence^24^. The strong advantages opened up by the RMPRS lie in the fact that by varying *B* one can efficiently control the light-mater interaction through the MP spectra. This opens a powerful platform for analysis of optoelectronic applications, such as tunable bandgaps enabling visible to near and middle infrared ultrafast lasers^25,26^, light emitters^27^, and biochemical sensors^28^. Among the further applications, it is important that TMD microcavities and polariton laser exhibit excellent stability being tunable by the applied magnetic field^29,30^.

## Methods

For the evaluation of the Raman intensity, $dI/d\Omega$, it is necessary to know the matrix elements for the electron-radiation, $\left\langle F\right| \hat{H}_{E-R}^{^{(\text {+})}}({\varvec{\sigma }}^\pm )\left| \Psi _{\mu _2}\right\rangle$, and electron-phonon, $\left\langle \Psi _{\mu _2}\right| \hat{H} _{E-P}\left| \Psi _{\mu _1}\right\rangle$ interaction, along with the structure of the Green’s function considering MP states. These quantities are derived by using the Green’s function method by casting the irreducible Feynman diagrams and solving the Dyson’s equation (see Supporting Information S2).

From the Raman selection rules, the renormalized state $\{\mu \}$ $\rightarrow$ *n*(*N*), hence, $G_{\mu }$ $\rightarrow$ $G_{n(N)}$. In TMD materials, the electron and hole effective masses are similar^17^. Under these considerations, the renormalization process for the EHP in a field *B* interacting with the optical phonon must be taken into account the contribution of both particles. Magneto-optical transitions between Landau levels have been observed in monolayer WSe$_2$^31,32^. The electron-hole correlation can be disregarded in case of high fields *B* and laser energies higher than the semiconductor gap. If the magnetic length $l_{B}$ is smaller than the exciton radius $r_{exc}$, the energetic space between the Landau levels will be greater than the exciton binding energy^33^. The relationship between $l_{B}$ and $r_{exc}$ depends on the excitonic states under consideration; for highly excited states the exciton energy dependence on *B* approaches the Landau levels. Typically, the energy separation in TMD between the 1S and 2S excitonic states is larger than the optical phonon energy, therefore the magneto-polaron effect is absent. For high values of *B*, the magneto-exciton energy shows a quasi-linear behavior with *B*^34^; this dependence is more pronounced in the high excited states. The same trend has been observed in bulk III–V materials and quantum wells. The resonant polaron energy $E_{n(N)}$, which appears in Eq. (2), is obtained by solving the transcendental equation

4 $$\begin{aligned} E_{n(N)}=\hbar \omega _{c}\left( N+\frac{1}{2}\right) +\alpha _{_\text {DP}}\hbar \omega _{c}\left( 1-\frac{D_{v}}{D_{c}}\right) \sum _{N^{\prime }=0}^{\infty }\bigg (\frac{1}{E-\Delta _{N^{\prime },N}-i\delta }-\frac{D_{v}/D_{c}}{E-\Delta _{N,N^{\prime }}-i\delta }\bigg ), \end{aligned}$$

where $\omega _{c}$ is the EHP cyclotron frequency and $\Delta _{N,N^{\prime }}=\hbar \omega _{A_{1}}+\hbar \omega _{c_e}(N+\frac{1}{2})+\hbar \omega _{c_h}(N^{\prime }+\frac{1}{2})$. The complex energy *E*=$\hat{\epsilon }_{n(N)}+\Gamma _{n(N)}$ with $\hat{\epsilon }_{n(N)}$ and $\Gamma _{n(N)}$ being the “dressed” EHP energy and its life-time broadening, respectively. The results (4) highlight that for a given Landau quantum number *N*, the MP quasi-particle is a combination of two independent electron or hole intravalley transitions described by the first and second term in Eq. (4). The presence of the hole (electron) Landau energy acts as a renormalized phonon energy, i.e. $\hbar \omega _{A_1} \rightarrow \hbar \omega _{A_1} +\hbar \omega _{c_e}(N+1/2)$ ($\hbar \omega _{A_1} \rightarrow \hbar \omega _{A_1} + \hbar \hbar \omega _{c_h}(N+1/2)$ ). Furthermore, the sum in Eq. (4) over $N^{\prime }$ indicates a mixing effect between different Landau levels in the conduction and valence bands. The contribution of the electron or hole states to the $E_{n(N)}$ depend on the $D_{c}, D_{v}$ and on the $m_e$, $m_h$ effective masses. Equation (4) together with the 2D MP energy levels disclosed in the Subsection EHP magneto-polaron energy, are the key methodological pillars of the present work.

### EHP magneto-polaron energy

Solving the transcendent Eq. (4), we obtain the EHP MP energies $\hat{\epsilon }_{n(N)}$ and the broadenings $\Gamma _{n(N)}$. For fixed *N* and *B* values, we label the new quantum states with *n* arranged in the increasing order of the energies $\hat{\epsilon }_{n(N)}$. Unlike a particle, where there is only one type of excited state, in the present case we are dealing with two different types of excitations, due to electrons and holes with energies $\hbar \omega _{e}^{(exc)}=\hbar \omega _{A_{1}}+ \hbar \omega _ {c_e}(p_e+1/2) +\hbar \omega _{c_h}(N +1/2)$ and $\hbar \omega _{h}^{(exc)}=\hbar \omega _{A_{1}}+ \hbar \omega _ {c_e}(N+1/2) +\hbar \omega _{c_h}(p_h +1/2)$ ($p_e$, $p_h$= 0, 1, 2,...), respectively. The frequencies $\omega _{c_e}^{(exc)}$ and $\omega _{c_h}^{(exc)}$ depend on the effective masses of the conduction and valence bands. Therefore, the slopes for the bare excited states are steeper, than those in the single-particle case. The EPI changes the symmetry imposed by the external field *B* on electrons and holes with Landau levels $N_e$ and $N_h$, establishing a new quantum state *n*. For a given *B*, the index *n* denotes the polaron ground state energy plus two sets of normalized excited states associated to the unperturbed energies $\hbar \omega _{e}^{(exc)}(p_e)$ and $\hbar \omega _{h}^{(exc)}(p_h)$. Therefore, there are two independent groups of avoiding crossings at the magnetic fields $B_e$ and $B_h$, as given by

5 $$\begin{aligned} \omega _{c_e}(B_e)=\frac{\omega _{A_{1}}}{N-p_e}\;\;\;\;\;; \;\;\; \;\;\; \omega _{c_h}(B_{h})=\frac{\omega _{A_1}}{N-p_h}; \;\;(p_e, p_h= 0, 1,\ldots , N-1). \end{aligned}$$

The coupling of the bare excited states, $\hbar \omega _{e}^{(exc)}$ and $\hbar \omega _{h}^{(exc)}$ with a certain Landau level leads to three excitation branches. We have two different types of MP resonances: one due to the coupling of two Landau levels in the conduction band via the EPI causing the occurrence of two branches, and another one due to the similar intervention of the holes in the valence band.

Figures 4 and 5 illustrate the EHP magneto-polaron spectra $\hat{\epsilon }_{n}$ with the quantum numbers $n=$ 2 and 3 as a function of the reduced cyclotron frequency $\omega _c /\omega _{A_1}$ for MoS$_2$ and WSe$_2$. The renormalized (uncoupled Landau levels) energies are indicated by solid (dashed) black lines and the bare electron (hole) excited states with $p_e$ ($p_h$) = $0, 1, \ldots ,N-1$ are shown by blue (red) open diamonds. In both materials, there are three branches involving the electron and hole cyclotron frequencies $\omega _{c_e}(B_e)$ and $\omega _{c_h}(B_h)$. In the case of MoS$_2$, the avoided crossings due to electron contributions occur at $\omega _c(B_e)/\omega _{A_1}$ = 0.6, 0.9 and 1.8, while for WSe$_2$, the gaps are clearly manifested for the valence band at $\omega _c(B_h)/\omega _{A_1}$ = 0.76, 1.14 and 2.29. This is a consequence of the ZO-deformation potential values, the relative contributions of the conduction and valences bands in the MP spectrum depend on the ratio $D_v/D_c$ (see Eq. (4)), which is 0.79 and 1.35 for MoS$_2$ and WSe$_2$, respectively. Therefore, the resonance effect due to the valence band will be more pronounced in WSe$_2$ than in the MoS$_2$ as it can be seen in Figs. 4 and 5.

From Fig. 4, it follows that for $n=$2 the energy of the lower branch is asymptotic to the bare electron excited state $\hbar \omega _{e}^{(exc)}(p_e=0)$. The intermediate $p_e=0$ and the third $p_h=0$ renormalized MP states approach the bare Landau level $N=$2. There is a mixing effect between the intermediate branch $p_e$=0 and the renormalized $p_h$=0 excited state. These two branches are asymptotic to the hole $\hbar \omega _{h}^{(exc)}(p_h=0)$ and the electron $\hbar \omega _{e}^{(exc)}(p_e=1)$ energies. The electron branch becomes the lower renormarlized energy of the next anticrossing at $\omega _c(B_e)/\omega _{A_1}$ $\approx$ 1.9. Here, the EPI couples the Landau level $N=2$ and two bare excited states with $p_e$ = 1, $p_h$ = 1. The same trend holds further for the MP quaisiparticle for *n* = 3. In Fig. 5, for the WSe$_2$ sample, a similar behavior holds true, with taking into account the exchange between the conduction and valence bands.

## Supplementary Information

Supplementary Information.

## Data Availability

The datasets used and/or analysed during the current study available from the corresponding author on reasonable request.

## References

[1] Ruf, T. *et al.* Resonant Raman scattering and piezomodulated reflectivity of InP in high magnetic fields. *Phys. Rev. B* **39**, 13378–13388. 10.1103/PhysRevB.39.13378 (1989). 10.1103/physrevb.39.13378 9948241

[2] Trallero-Giner, C., Santiago-Pérez, D. G. & Fomin, V. M. New magneto-polaron resonances in a monolayer of a transition metal dichalcogenide. *Sci. Rep.* **13**, 292. 10.1038/s41598-023-27404-x (2023). 36609670 10.1038/s41598-023-27404-x PMC9822968

[3] Ruf, T., Phillips, R. T., Trallero-Giner, C. & Cardona, M. Resonant magneto-Raman scattering in GaAs. *Phys. Rev. B* **41**, 3039–3047. 10.1103/PhysRevB.41.3039 (1990). 10.1103/physrevb.41.3039 9994074

[4] López, V., Comas, F., Trallero-Giner, C., Ruf, T. & Cardona, M. Resonant electron-phonon coupling: Magnetopolarons in inp. *Phys. Rev. B* **54**, 10502–10507. 10.1103/PhysRevB.54.10502 (1996). 10.1103/physrevb.54.10502 9984845

[5] Heiman, D. *High Magnetic Fields in The Physics of Semiconductors* (World Scientific, 1995).

[6] Tan, T., Jiang, X., Wang, C., Yao, B. & Zhang, H. 2D material optoelectronics for information functional device applications: Status and challenges. *Adv. Sci.* **7**, 2000058. 10.1002/advs.202000058 (2020). 10.1002/advs.202000058 PMC7284198 32537415

[7] Cheng, Z. *et al.* 2D materials enabled next-generation integrated optoelectronics: From fabrication to applications. *Adv. Sci.* **8**, 2003834. 10.1002/advs.202003834 (2021). 10.1002/advs.202003834 PMC8188205 34105275

[8] Tian, H. *et al.* Optoelectronic devices based on two-dimensional transition metal dichalcogenides. *Nano Res.* **9**, 1543–1560. 10.1007/s12274-016-1034-9 (2016).

[9] McDonnell, S. J. & Wallace, R. M. Atomically-thin layered films for device applications based upon 2D TMDC materials. *Thin Solid Films* **616**, 482–501. 10.1016/j.tsf.2016.08.068 (2016).

[10] Johnson, E. J. & Larsen, D. M. Polaron induced anomalies in the interband magnetoabsorption of InSb. *Phys. Rev. Lett.* **16**, 655–659. 10.1103/PhysRevLett.16.655 (1966).

[11] Trallero-Giner, C., Santiago-Pérez, D. G., Vasilevskiy, M. I. & Marques, G. E. Rydberg excitons and doubly resonant Raman scattering in transition-metal dichalcogenides. *J. Phys. Chem. C* **128**, 210–217. 10.1021/acs.jpcc.3c06303 (2024).

[12] Belitsky, V. I., Trallero-Giner, C. & Cardona, M. Magnetopolaron effect in one-phonon resonant Raman scattering from bulk semiconductors: Deformation potential. *Phys. Rev. B* **48**, 17861–17866. 10.1103/PhysRevB.48.17861 (1993). 10.1103/physrevb.48.17861 10008417

[13] Belitsky, V. I., Trallero-Giner, C. & Cardona, M. Magnetopolaron effect in one-phonon resonant Raman scattering from bulk semiconductors: Fröhlich interaction. *Phys. Rev. B* **49**, 11016–11020. 10.1103/PhysRevB.49.11016 (1994). 10.1103/physrevb.49.11016 10009946

[14] Trallero-Giner, C., Menéndez-Proupin, E., Morell, E. S., Pérez-Álvarez, R. & Santiago-Pérez, D. G. Phenomenological model for long-wavelength optical modes in transition metal dichalcogenide monolayer. *Phys. Rev. B* **103**, 235424. 10.1103/PhysRevB.103.235424 (2021).

[15] Zhang, X. *et al.* Phonon and Raman scattering of two-dimensional transition metal dichalcogenides from monolayer, multilayer to bulk material. *Chem. Soc. Rev.* **44**, 2757–2785. 10.1039/C4CS00282B (2015). 25679474 10.1039/c4cs00282b

[16] Trallero-Giner, C., Cantarero, A., Cardona, M. & Mora, M. Impurity-induced resonant Raman scattering. *Phys. Rev. B* **45**, 6601–6613. 10.1103/PhysRevB.45.6601 (1992). 10.1103/physrevb.45.6601 10000420

[17] Kormányos, A. *et al.* kÂ.p theory for two-dimensional transition metal dichalcogenide semiconductors. *2D Mater.* **2**, 022001. 10.1088/2053-1583/2/2/022001 (2015).

[18] Huang, Z., Zhang, W. & Zhang, W. Computational search for two-dimensional MX semiconductors with possible high electron mobility at room temperature. *Materials* **9**, 1–10 (2016). 10.3390/ma9090716 PMC5457095 28773835

[19] Kaasbjerg, K., Thygesen, K. S. & Jacobsen, K. W. Phonon-limited mobility in -type single-layer Mos from first principles. *Phys. Rev. B* **85**, 115317. 10.1103/PhysRevB.85.115317 (2012).

[20] Jin, Z., Li, X., Mullen, J. T. & Kim, K. W. Intrinsic transport properties of electrons and holes in monolayer transition-metal dichalcogenides. *Phys. Rev. B* **90**, 045422. 10.1103/PhysRevB.90.045422 (2014).

[21] Iikawa, F., Ruf, T. & Cardona, M. Hot luminescence and Landau-level fine structure in bulk GaAs. *Phys. Rev. B* **43**, 4849–4855. 10.1103/PhysRevB.43.4849 (1991). 10.1103/physrevb.43.4849 9997856

[22] Goryca, M. *et al.* Revealing exciton masses and dielectric properties of monolayer semiconductors with high magnetic fields. *Nat. Commun.* **10**, 4172. 10.1038/s41467-019-12180-y (2019). 31519909 10.1038/s41467-019-12180-y PMC6744484

[23] Cassabois, G., Valvin, P. & Gil, B. Hexagonal boron nitride is an indirect bandgap semiconductor. *Nat. Photon.* **10**, 262–266. 10.1038/nphoton.2015.277 (2016).

[24] Paradisanos, I. *et al.* Efficient phonon cascades in WSe monolayers. *Nat. Commun.* **12**, 538. 10.1038/s41467-020-20244-7 (2021). 33483475 10.1038/s41467-020-20244-7 PMC7822848

[25] Xia, H. *et al.* Few-layer MoS grown by chemical vapor deposition as a passive Q-switcher for tunable erbium-doped fiber lasers. *Photon. Res.* **3**, A92–A96. 10.1364/PRJ.3.000A92 (2015).

[26] Liu, W. *et al.* Nonlinear optical properties of WSe and MoSe films and their applications in passively Q-switched erbium doped fiber lasers. *Photon. Res.* **6**, C15–C21. 10.1364/PRJ.6.000C15 (2018).

[27] Cadiz, F. *et al.* Excitonic linewidth approaching the homogeneous limit in MoS-based van der Waals heterostructures. *Phys. Rev. X* **7**, 021026. 10.1103/PhysRevX.7.021026 (2017).

[28] Late, D. J. *et al.* Sensing behavior of atomically thin-layered MoS transistors. *ACS Nano* **7**, 4879–4891. 10.1021/nn400026u (2013). 23713986 10.1021/nn400026u

[29] Zhao, J. *et al.* Exciton polariton interactions in Van der Waals superlattices at room temperature. *Nat. Commun.* **14**, 1512. 10.1038/s41467-023-36912-3 (2023). 36932078 10.1038/s41467-023-36912-3 PMC10023709

[30] Kavokin, A. *et al.* Polariton condensates for classical and quantum computing. *Nat. Rev. Phys.* **4**, 435–451. 10.1038/s42254-022-00447-1 (2022).

[31] Wang, Z., Shan, J. & Mak, K. F. Valley- and spin-polarized landau levels in monolayer WSe. *Nat. Nanotechnol.* **12**, 144–149. 10.1038/nnano.2016.213 (2017). 27798606 10.1038/nnano.2016.213

[32] Li, J. *et al.* Spontaneous valley polarization of interacting carriers in a monolayer semiconductor. *Phys. Rev. Lett.* **125**, 147602. 10.1103/PhysRevLett.125.147602 (2020). 33064502 10.1103/PhysRevLett.125.147602

[33] Stier, A. V. *et al.* Magnetooptics of exciton Rydberg states in a monolayer semiconductor. *Phys. Rev. Lett.* **120**, 057405. 10.1103/PhysRevLett.120.057405 (2018). 29481196 10.1103/PhysRevLett.120.057405

[34] Ly, D.-N. *et al.* Retrieval of material properties of monolayer transition metal dichalcogenides from magnetoexciton energy spectra. *Phys. Rev. B* **107**, 205304. 10.1103/PhysRevB.107.205304 (2023).

