# Supplementary material for: Raman scattering owing to magneto-polaron states in monolayer transition metal dichalcogenides
Source: Sci Rep. 2024 Jun 4;14:12857. doi: 10.1038/s41598-024-63179-5 (PMC11637126; doi:10.1038/s41598-024-63179-5)
Supplement: Supplementary file 1 — Supplementary Information. [file 41598_2024_63179_MOESM1_ESM.pdf]

Supplementary Information

Raman Scattering owing to Magneto-Polaron States in Monolayer Transition Metal Dichalcogenides

C. Trallero-Giner,[1] D. G. Santiago-Pérez,[2] D. V. Tkachenko,[3] G. E. Marques,[1] and V. M. Fomin[4,5]

[1]*Departamento de Física, Universidade Federal de São Carlos, 13.565-905, São Carlos, São Paulo, Brazil*

[2]*Universidad Autónoma del Estado de Morelos, Ave. Universidad 1001, CP 62209, Cuernavaca, Morelos, México*

[3]*Pridnestrovian State University, 25 October str., 128, MD-3300, Tiraspol, Republic of Moldova*

[4]*Institute for Emerging Electronic Technologies (IET), Leibniz Institute for Solid State and Materials Research (IFW) Dresden, Helmholtzstraβe 20, D-01069 Dresden, Germany*

[5]*Faculty of Physics and Engineering, Moldova State University, str. A. Mateevici 60, MD-2009, Chiău, Republic of Moldova*

S1. Electron-radiation and electron-phonon interactions

The free EHP energy in a field B is given by $\epsilon_\mu = \hbar\omega_{c_e}(N_e + 1/2) + \hbar\omega_{c_h}(N_h + 1/2)$, where ω_{c_e} (ω_{c_h}) is the electron (hole) cyclotron frequency. The corresponding wave function can be cast as $|\Psi_\mu\rangle = |\psi_{\mu_e}\rangle|u_c\rangle|\psi_{\mu_h}\rangle|u_h\rangle$, where $|u_c\rangle$ and $|u_v\rangle$ are the Bloch functions for the 2D conduction and valence bands at the K or $K^{'}$-point of the BZ. The electron and hole wave functions $|\psi_{\mu_i}\rangle$ $(i = e, h)$ are $\psi_{\mu_i} = S^{-1/2}e^{ik_{iy}y_i}\varphi_{N_i}(x)f(z_i)$, where φ_N are the harmonic oscillator functions and $f(z_i)$ is the Gaussian function $f(z_i) = (\sqrt{\pi}b)^{-1/2}e^{-z^2/2b^2}$. The electron-radiation matrix element for the K or $K^{'}$-point of the BZ is [1]

$$\left\langle \mu_1 | H_{E-R}^{(-)} | I \right\rangle = \mp \frac{e}{m_0}\sqrt{\frac{4\pi}{\hbar\omega\eta^2}}\frac{at}{\sqrt{V}}\delta_{N_e,N_h}\delta_{k_{hy},k_{ey}\pm\kappa_y}\boldsymbol{\sigma}\cdot\boldsymbol{\sigma}^{\pm} \;, \tag{S1}$$

where m_0 and e are the free electron mass and charge, ω, $\boldsymbol{\kappa}$ and $\boldsymbol{\sigma}$ are the photon frequency, the wave vector and the polarization, respectively, a is the lattice constant, t is the hopping parameter [2, 3], $\boldsymbol{\sigma}^{\pm} = (\boldsymbol{e_x} \pm i\boldsymbol{e_y})/\sqrt{2}$, $\boldsymbol{e_x}$ and $\boldsymbol{e_y}$ are unit vectors along the corresponding axes. In the calculation we consider the EHP confined to the 2D plane, i.e. $b \to 0$. The same result holds true for $\left\langle F | H_{E-R}^{(+)} | \mu_2 \right\rangle$.

For a ML TMD, the symmetry group of the Γ-point normal mode is D_{3h} [4]. Optical phonon modes with irreducible representations A_1(ZO) vibrating out-of-plane and E'(LO) oscillating in-plane [5], couple EHP states via intravalley EPI at the K or $K^{'}$-valleys [6]. Therefore, two main mechanisms have to be considered for the first-order Raman scattering in backscattering configurations with parallel polarization: the short-range ZO-homopolar DP and the long-range LO-phonon PF interactions [7]. Assuming that the in-plane phonon wave vector $\boldsymbol{q} = \boldsymbol{0}$, the matrix elements are: for the electron-hole DP

$$\langle \Psi_{\mu_2} | \, \hat{H}_{E-P}^{DP} \, | \Psi_{\mu_1} \rangle = \left(\frac{\hbar}{2\rho_m A N_c \omega_{A_1}} \right)^{1/2} [D_c - D_v] \, \delta_{N_e,N_h}\delta_{k_{hy},k_{ey}} \tag{S2}$$

and for the PF Hamiltonian

$$\langle \Psi_{\mu_2} | \, \hat{H}_{E-P}^{PF} \, | \Psi_{\mu_1} \rangle = \frac{-i\mathbb{G}_{Ph}}{\sqrt{N_c}(1 + r_0 q)} \left[\langle \Psi_{\mu_2} | \, e^{i\boldsymbol{q}\cdot\boldsymbol{\rho_e}} \, | \Psi_{\mu_1} \rangle - \langle \Psi_{\mu_2} | \, e^{i\boldsymbol{q}\cdot\boldsymbol{\rho_h}} \, | \Psi_{\mu_1} \rangle \right] |_{\boldsymbol{q}=0} = 0 \;. \tag{S3}$$

S2. Dyson's equation

The resonant polaron energy $E_n(N)$ in Eq. (2) is obtained by solving the Dyson's equation for $G_{n(N)}(E)$. It is possible to show that $G_n(E) = G_N^0/[1 - G_N^0 S(E, N)]$, with G_N^0 the unperturbed Green's function at T= 0 K, and $S(E, N)$ the self-energy due to the EHP coupling to the optical phonon modes. The main Feynman diagrams in the lowest order of the electron-phonon coupling constant contributing to $S(E, N)$ are represented in Fig. 1. Employing the electron-phonon Hamiltonian, the contribution of the Feynman diagrams of Fig. 1 to the self-energy can be cast

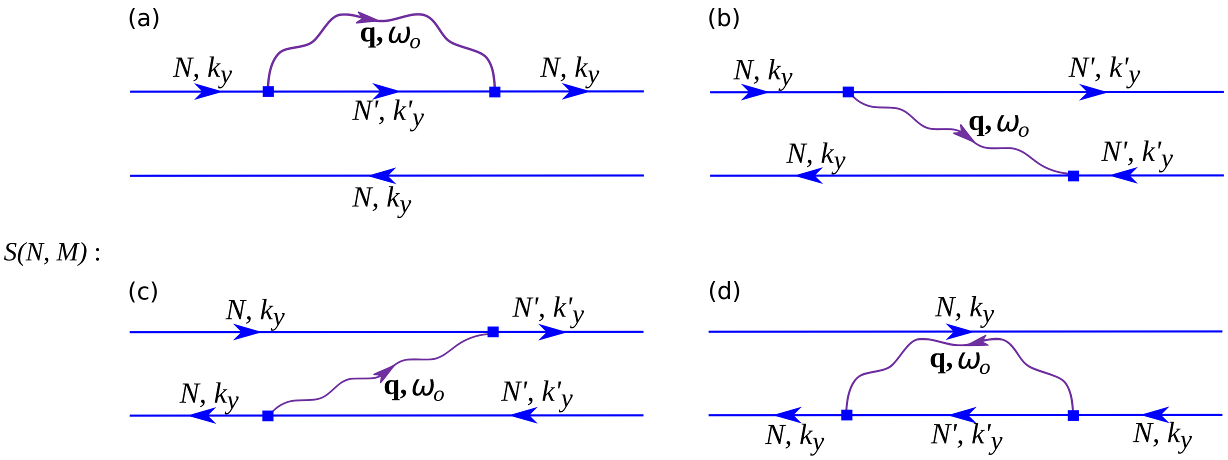

FIG. 1. Four irreducible Feynman diagrams contributing to the EHP self-energy $S_E(N,M)$ due to the optical phonon ω_o coupling to: electron-electron (a), electron-hole (b), hole-electron (c), and hole-hole (d) states.

as

$$S(E,N) = \frac{1}{l_c^2}\sum_{N'}\Bigg[\frac{\overline{T}^{(e-e)}_{N',N}+\overline{T}^{(e-h)}_{N',N}}{E-\hbar\omega_o-\hbar\omega_{c_e}(N'+1/2)-\hbar\omega_{c_h}(N+1/2)-i\delta}+ \\ +\frac{\overline{T}^{(h-h)}_{N',N}+\overline{T}^{(e-h)*}_{N',N}}{E-\hbar\omega_o-\hbar\omega_{c_e}(N+1/2)-\hbar\omega_{c_h}(N'+1/2)-i\delta}\Bigg], \quad \text{(S4)}$$

where $\overline{T}^{(i-j)}_{N',N}$ is the matrix element coupling the particles i and j as given by

$$\overline{T}^{(i-j)}_{N',N} = \frac{S}{2\pi}\int_0^\infty dQ C_Q^{(i)*}C_Q^{(j)}T_{N',N}(Q)\,, \quad \text{(S5)}$$

$C_Q^{(i)}$ is the electron-phonon coupling constant, and the function $T_{N',N}(Q)$ is reported in Ref. [6]. In principle, both types of interactions, DP for the out-of-plane ZO-homopolar mode and PF for the in-plane LO-phonon, have to be considered.

For the DP interaction, when $C_Q^{i)} = C^{i)} = [\hbar/(2\rho_m AN_c\omega_{A_1})]^{1/2} D_i$ $(i=c,v)$, the sum is

$$\overline{T}^{(e-e)}_{N',N}+\overline{T}^{(e-h)}_{N',N} = \left(D_c^2 - D_cD_v\right)\frac{\hbar}{2\rho_m AN_c\omega_{A_1}}\,. \quad \text{(S6)}$$

The same result is achieved for $\overline{T}^{(h-h)}_{N',N}+\overline{T}^{(e-h)^*}_{N',N}$ with $(e)\leftrightarrow(h)$. Collecting the results of (S5) and (S6), we obtain

$$S^{(\mathrm{DP})}(E,N) = \alpha_{\mathrm{DP}}\hbar\omega_{c_e}\hbar\omega_{A_1}\left(1-D_v/D_c\right)\times \\ \sum_{N'}\Bigg[\frac{1}{E-\hbar\omega_{A_1}-\hbar\omega_{c_e}(N'+1/2)-\hbar\omega_{c_h}(N+1/2)-i\delta} - \\ \frac{D_v/D_c}{E-\hbar\omega_{A_1}-\hbar\omega_{c_e}(N+1/2)-\hbar\omega_{c_h}(N'+1/2)-i\delta}\Bigg], \quad \text{(S7)}$$

with $\alpha_{\mathrm{DP}} = m_eD_c^2//(4\pi\rho_m\hbar^2\omega_{A_1}^2)$.

For the PF interaction, $\overline{T}^{(e-e)}_{N',N}+\overline{T}^{(e-h)}_{N',N}=0$ and $\overline{T}^{(h-h)}_{N',N}+\overline{T}^{(e-h)*}_{N',N}=0$. This result is a consequence of considering the backscattering configurations and assuming a strictly 2D PF Hamiltonian. From Eq. (S7), when the hole effective mass $\to\infty$ and consequently $D_v=0$, the result of Ref. [6] is recovered. The pole of the renormalized Green's function provides the complex-valued MP energy $E_{n(N)}(B)$, while $\Im\{E_{n(N)}\}=\Gamma_{n(N)}$ its lifetime broadening. Figure 2 represents the dependence of Γ_n on B in ML MoS_2 and WSe_2 for the MP states $n=2$. The resonant part of the dressed Landau level $N=2$ and four renormalized electron and hole excited states (p_e, $p_h=0$, 1) are clearly manifested at the avoided crossing points.

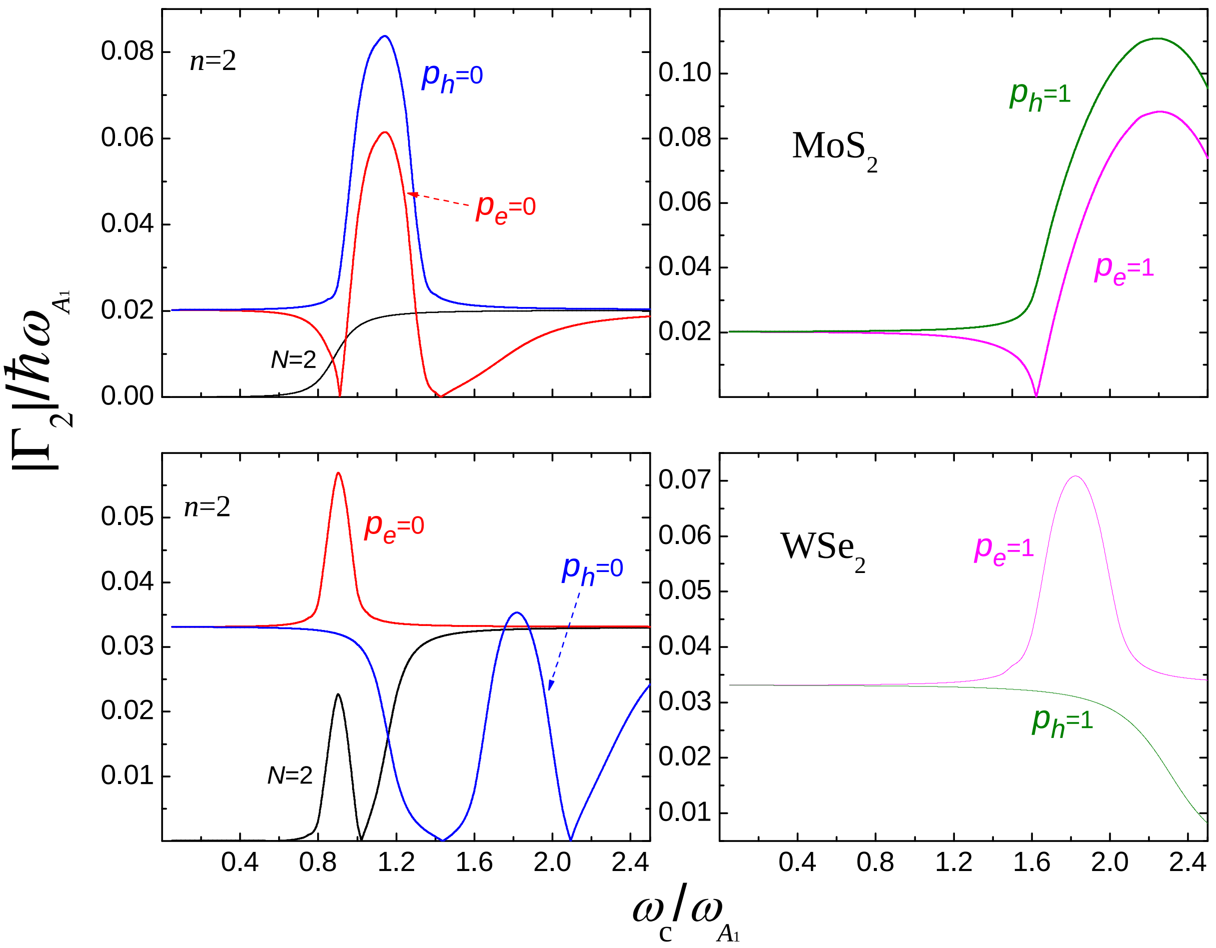

FIG. 2. Lifetime broadening, $|\Gamma_2|$ as a function of the relative cyclotron energy ω_c/ω_{A_1} in MoS_2 (upper panels) and WSe_2 (lower panels) for the MP states n=2. The dressed Landau level N=2, the electron (hole) renormalized excited states p_e (p_h) =0 and p_e (p_h) =1 are shown by black, red (olive) and magenta (olive) lines, respectively.

[1] C. Trallero-Giner, D. G. Santiago-Pérez, M. I. Vasilevskiy, and G. E. Marques, Rydberg excitons and doubly resonant raman scattering in transition-metal dichalcogenides, The Journal of Physical Chemistry C **128**, 210 (2024).

[2] D. Xiao, G.-B. Liu, W. Feng, X. Xu, and W. Yao, Coupled Spin and Valley Physics in Monolayers of MoS_2 and Other Group-VI Dichalcogenides, Phys. Rev. Lett. **108**, 196802 (2012).

[3] C. Zhang, H. Wang, W. Chan, C. Manolatou, and F. Rana, Absorption of light by excitons and trions in monolayers of metal dichalcogenide MoS_2: Experiments and theory, Phys. Rev. B **89**, 205436 (2014).

[4] N. Scheuschner, R. Gillen, M. Staiger, and J. Maultzsch, Interlayer resonant Raman modes in few-layer MoS_2, Phys. Rev. B **91**, 235409 (2015).

[5] X. Zhang, X.-F. Qiao, W. Shi, J.-B. Wu, D.-S. Jiang, and P.-H. Tan, Phonon and Raman scattering of two-dimensional transition metal dichalcogenides from monolayer, multilayer to bulk material, Chem. Soc. Rev. **44**, 2757 (2015).

[6] C. Trallero-Giner, D. G. Santiago-Pérez, and V. M. Fomin, New magneto-polaron resonances in a monolayer of a transition metal dichalcogenide, Scientific Reports **13**, 292 (2023).

[7] C. Trallero-Giner, D. G. Santiago-Pérez, M. I. Vasilevskiy, and G. E. Marques, Rydberg Excitons and Doubly Resonant Raman Scattering in Transition-Metal Dichalcogenides, The Journal of Physical Chemistry C **128**, 210 (2024).
